# Indents

**Published:** 1920-12

**Authors:** 


### INDENTS

**☞ Brief Articles of Technical or Scientific Value will receive notice and comment. Contributions invited.**

---

Strength of the Labial, Buccal and Lip Functions. As to muscular development for the correction of unbalanced muscles, I think it would be of great assistance in Class II, Division I cases. I believe such cases are all preventable if we could prescribe the use of some musical wind instrument at an early age that would develop the labial muscles, and I do not think under such circumstances we would ever have a case of protruding upper centrals and laterals with these children who exhibit that tendency at the beginning, if they were taught and compelled to practice on the clarinet, slide trombone, cornet, etc. I have worked for patients who have practiced on such instruments from childhood, and the lips were so rigid it was difficult to keep them out of the way of the work. I noticed it in general practice especially, and in one case of correction of malocclusion where the central incisors were tipped lingually, it was due to over-development of the labial muscle, and I doubt if we can retain the teeth in normal position on account of the over-development of the upper lip. The same thing occurs in hair-lip that has been operated on. I undertook the correction of one or two of these cases. Never again! The contractile tissue will force the central incisors lingually in spite of your efforts.—W. Cavanagh, in *Pacific Gazette.*

---

What Kind of Bridge Shall We Make? Dr. M. L. Ward says that many refrain from doing bridge work rather than make an effort to improve the defective features and retain the good ones. He urges that those who are afraid to do bridge work resume their efforts to place the right kind of a bridge in the right place rather than refrain from doing bridge work. At the outset he emphatically states that "an effort to do justice to a general practice with only one type of such (bridge) work is as nonsensical as to try to serve the best interests of a general practice with one filling material, one type of surgical operation, one procedure in orthodontia, one in prosthodontia, one method of handling pyorrhea,, or one way to treat the pulps of teeth. There should be no such thing in the mind of an advanced thinker as the best type or form of bridge work to apply. The thought that should be foremost in the mind of such a man is to have such a variety of types so thoroughly mastered that he is able to apply the one which seems most suitable for the case in question, and in many instances to combine two or more types in order that the maximum may be rendered and the minimum of undesirable features presented." He then calls attention to three principal changes which are being made in the practices of the more progressive bridge workers, namely:

1. Reduction in the number of full crowns used.

2. As a result of this a much smaller number of teeth are devitalized.

3. Cements which are antiseptic are used for the retention of crowns and bridges.

After reviewing the relative merits of fixed and removable forms of bridges, he says: "I take this opportunity to record myself as saying that from now on the chances for infection at either the apex or cervix of the roots of teeth shall be the first thing considered when I am consulted about bridge work. Much as I would like to place a good removable bridge in the mouths of some patients from the standpoint of mastication, and sometimes appearance, I shall have to be shown that what I am to accomplish for the patient must completely overbalance the destruction to the teeth necessary to place the bridge, which often increases the liability to apical and cervical infection. * * * We should in my opinion adopt what appears to be a more modern tendency, viz., cut down the number of full crowns and thereby cut down the number of devitalizations, even if we are obliged to cut down the amount of both fixed and of removable bridge work. In our practice at the present time we are using almost exclusively the red and black copper cements and the cuprous iodide in the form of copper zinc for all forms of crowns. In the cases that look to be difficult to be kept clean, we are adopting the jet black one made by Ames and Caulk, and the red ones made by Fleck and Caulk. In cases where a decidedly red or black color would be objectionable, we are using the copper zinc product, about equal parts of copper and zinc with crown and bridge powder."—*Journal N. D. Assn.*

---

SUGAR DIET AND DENTAL CARIES. Whether we eat starch or sugar, it does not pass the wall of the intestinal tract until it has been converted into some kind of sugar. It is essential to differentiate between the kinds of sugar. Before either can get through the intestinal tract it must be converted into sugar, and the kind of sugar itself converted is the same in each instance; therefore the distinction between starch and sugar has ceased to exist once the intestinal wall has been passed.

For the promotion of dental decay, it is necessary that there should be a certain amount of carbohydrate accessible to the organisms which produce lactic acid and remain comparatively quiet for a certain length of time. When sugar or starch as sugar gets into the blood stream, it is stored in one or two places, either in the liver or muscles, and it is fed from there under varying circumstances to the blood stream.

We have learned a good deal in the course of the last few years about blood sugar, and the circumstances that determine an increase or decrease in the amount of sugar in the blood. We have found that there are circumstances under which it is considerably greater than it is under other circumstances.

The question arises, what is the relation between those connections and dental decay? There is, for instance, a relative sugar starvation in pregnancy; in other words, that there is less than the normal amount of sugar under these circumstances. You would expect, therefore, there would be a diminished amount of tooth decay during pregnancy. Is that true? Clinically, if we are careful in our observations, the amount of dental decay is increased in diabetes. Those are two illustrations. As a matter of fact, what we are interested in is the amount of sugar excreted from the blood into the saliva, or, in other words, a quantitative determination of one sort of sugar or another in the saliva of blood origin under certain circumstances, and can that be connected with tooth decay? I do not believe there is any logic in the idea that the sugar which is in the blood stream can be the basis for the formation by lactic acid bacilli of lactic acid in the blood stream, and that they act upon the teeth to bring about decay. I am quite prepared to believe that as we begin to learn more and more about the amount of sugar in the blood stream in various clinical conditions, that as we begin to learn more and more about the amount of sugar in various excretions and secretions, we may pile up data that may serve as a basis for a conclusion, because I would not be surprised at all if the conclusion is correct, because about this I am sorry there is not an actual parallelism between the amount of bacteria capable of producing decay in the mouth and the amount of decay produced; or, in other words, that there are times when it varies one with the other; the two curves depart from each other very materially.—Dr. Wm. A. Evans, in *Journal N. D. A.* for June.

SELECTION OF PATIENTS. Use the same care in selecting patients, as patients do in selecting dentists. This practice will keep you out of lots of grief and end up with patients who are in harmony with you and for whom you can do your very best. If you don't do this you will get into your practice many who are not satisfactory patients, and these patients keep the more desirable from getting your service. No one has a right to come to you for service without first making preparation for credit, if credit is wanted.

When you make an appointment, give to the patient a card on which the appointed time is indicated and the terms under which it is made. This relieves the patient of the necessity of remembering the appointment. It enables your card to enter the home where others will see it, and as the card carries the conditions under which the appointment is made and can be broken, enables you to set up a fee for broken appointments without protest.

MRS.................................

has an appointment with

DR. JOHN B. SMITH

10 A. M., Saturday, June 12, 1920.

If for any reason you can not keep this appointment, a notice of twenty-four hours must be given; otherwise a charge will be made for the time reserved.

PHONE —.

This can be enclosed in a small envelope and mailed when used for patients who are to come in at definite periods for prophylactic work. Just the moment dentists run their own affairs in their office, just that soon will the conduction of that office become a pleasure. Arrange definite plan of operation and adhere to it for all, then you will be happy in the practice of dentistry. Under the present unusual conditions when all lines of business are changing their plans of conducting their affairs, it is the psychological time for dentists to decide the ideal plan and follow it.—Harry J. Bosworth, Chicago, *Digest*.

